# Percutaneous Muscular Ventricular Septal Defect Closure with 2D Transthoracic Echocardiography: Can We Sufficiently Visualize It?

**DOI:** 10.5152/eurasianjmed.2021.20131

**Published:** 2021-06

**Authors:** Deniz Mutlu, Konstantinos Marmagkiolis, Cezar A. Iliescu2, Ismail Ates, Mehmet Cilingiroglu

**Affiliations:** 1 Department of Cardiology, İstanbul University-Cerrahpasa, Cerrahpasa Faculty of Medicine, İstanbul Turkey; 2 Department of Cardiology, University of Texas, MD Anderson Cancer Center, Houston, TX ABD; 3 Department of Cardiology, Premier Heart and Vascular Group, Tampa, FL, USA; 4 Department of Cardiology, Bahçeşehir University School of Medicine, İstanbul, Turkey; 5 Department of Cardiology, Medical Park Hospital, Antalya, Turkey; 6 Department of Cardiology, University of Texas Health Sciences Center, San Antonio, TX, USA

**Keywords:** Ventricular septal defect, Congenital heart defect, Endovascular technique, Transthoracic Echocardiography

## Abstract

Ventricular septal defect (VSD) is one of the most common congenital heart diseases worldwide today. Although the majority close spontaneously, transcatheter VSD closure is a common option for symptomatic patients with suitable anatomy in adult age. Although transesophageal echocardiography (TEE) and intracardiac echocardiography are the most common imaging modalities for the procedure, in patients with poor TEE images, Transthoracic echocardiography (TTE) can be used as a reliable alternative. Here we present an adult patient with pulmonary hypertension associated with a muscular VSD which was closed percutaneously using 2-dimensional TTE because of poor TEE images.

## Introduction

Ventricular septal defect (VSD) is one of the most common congenital heart diseases worldwide today. 1 Although it is more frequently encountered in the pediatric age, it accounts for approximately 10% of adult congenital heart diseases due to the spontaneous closure of the defect.^1^ There may be a single or multiple defects, or complicated by subpulmonary stenosis, pulmonary hypertension (PHT), and/or aortic regurgitation.^2^ Among them PHT is crucial because it can lead to right heart volume and pressure overload secondary to left-to-right ventricular shunt through the VSD. Here we present an adult patient with mild PHT and significant shunt associated with a muscular VSD which was closed percutaneously using only 2-dimensional transthoracic echocardiography (TTE) because of poor transesophageal echocardiography (TEE) images.

## Case Description

**Figure 1:**
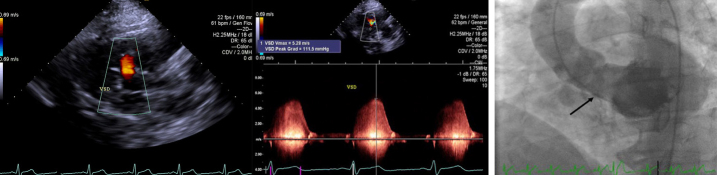
(top left) Transthoracic echocardiogram (TTE) demonstrates muscular ventricular septal defect (VSD) in the parasternal long axis view, (top right) continuous wave Doppler probing demonstrates VSD in TTE, (bottom middle) left ventriculogram demonstrates a small muscular VSD in the left anterior oblique projection.

A 39 years-old man with known muscular VSD since birth was admitted with exertional dyspnea, and several episodes of frank syncope. After thorough evaluation, he was selected for transcatheter closure of VSD due to a significant left-to-right shunt according to the 2018 ACC/AHA guideline.^3^ An informed consent was taken according to the Helsinki Decleration. His TTE has demonstrated (ACUSON SC2000 Prime Ultrasound System, Siemens Healthcare GmbH, Erlangen, Germany) that the left ventricular ejection fraction (LVEF) was 55%–60%, there was a 1 cm diameter muscular VSD (Figure 1), and a pulmonary artery systolic pressure of 48 mm Hg. Preprocedure electrocardiography showed a normal sinus rhythm with a right bundle branch block and voltage criteria for left ventricular hypertrophy. His lab results were unremarkable with the normal basic metabolic panel and complete blood count. Right heart catheterization was performed and showed a mild PHT and significant left-to-right shunt. (Mean pulmonary artery pressure: 26 mm Hg, Qp/Qs: 1.6, left-to-right shunt level: 3 liters per minute) (Table 1). Subsequently, a left ventriculogram was performed in the left anterior oblique (LAO) projection confirming a small muscular VSD. (Figure 1) The coronary arteries were normal and LVEF was 55%.

**Figure 2:**
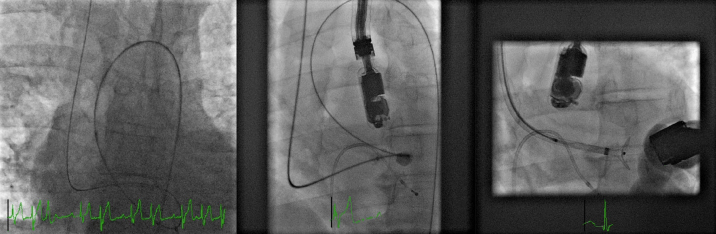
(left) Fluoroscopic image demonstrates a glidewire loop which crosses and snares the VSD, then externalizes to the right internal jugular vein. Middle: balloon sizing of the VSD. Right: the closure of the VSD with 16 mm Amplatzer VSD occluder.

**Figure 3:**
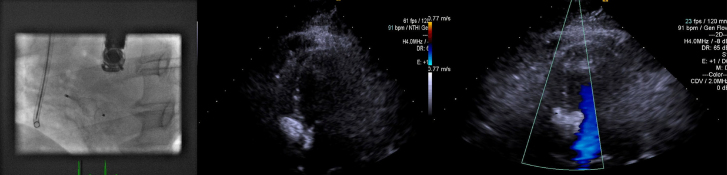
(top left) Fluoroscopic image demonstrates the confirmed position of the device, (top right) transthoracic echocardiogram (TTE) demonstrates the confirmed position of the device, (bottom middle) postoperative 30th day control TTE demonstrates the successful closure.

After moderate sedation was induced by the anesthesiologist, the procedure started with a JR4 catheter (Cordis Corporation, Milpitas, CA, USA) which was initially placed at the left ventricle (LV) after crossing the aortic valve and a glidewire (Terumo International, Shibuya, Tokyo, Japan) which crossed the VSD and was snared and externalized to the right internal jugular vein to complete a loop. (Figure 2) The glidewire was exchanged with the Amplatzer guidewire (St. Jude Medical Inc., Saint Paul, Minnesota, USA) through the JR4 catheter, followed by balloon sizing. At first, TEE was tried to obtain an optimal image to visualize the VSD. Due to the inability to optimize and visualize the correct level and a massive amount of secretion in the esophagus, TTE was selected to proceed with the procedure. The defect was found to be suitable for closure with a 16 mm Amplatzer VSD occluder by using the apical, 4-chamber position (A4C) of the TTE and the LAO projection of the flouroscopy. (Figure 2) The VSD was successfully closed after 2 unsuccessful attempts. The configuration of the device was checked by TTE and fluoroscopy, and the position of the device was found to be satisfactory. (Figure 3) A control TTE was performed one month after the procedure, and it showed a successful closure without tilting or displacement of the device.

## Discussion

There are 5 types of VSD: the infundibular, which is located beneath the aortic and pulmonary valves; membranous defects; the inlet defects; muscular defects; and the Gerbode defect.^2^ The most common one is membranous defect which is defined as the deficiency of the membranous septum. The inlet defects occur around the atrioventricular canal, beneath the mitral and tricuspid valves. When it comes to muscular defects, the incidence is approximately 5%–20% of all types of VSD bordered only by muscle in the trabecular septum.^2^ Our patient had this type of defect. The least common one is the Gerbode defect, which is defined as the deficiency of the septum, separating the LV from the right atrium (RA) and causing LV to RA shunting.

Although there are numerous associated defects concurrent with VSD in children (atrial septal defect, patent ductus arteriosus, transposition of great arteries, Tetralogy of Fallot, etc.), the majority of adult patients present with an isolated defect.^2^,^4^ In addition, there are acquired causes of VSD, which can occur as a complication of an acute myocardial infarction, a surgical or transcatheter aortic valve replacement or septal myectomy in patients with hypertrophic cardiomyopathy (HCMP).

The transcatheter VSD closure (TVSDC) is a novel option for muscular and certain membranous VSDs with suitable anatomy. For instance, the location of the defect must be remote from aortic and tricuspid valves with a sufficient rim of tissue.^5^ The first successful closure of VSD was performed in 1988.^5^ There is an important learning curve for the successful closure of VSD. Complication rates have decreased considerably with experience. The Amplatzer VSD occluder (St. Jude Medical, St. Paul, USA) is the most widely used device with the 98.1% technical success.^4^,^5^ Although TEE and intracardiac echocardiography (ICE) are the most common imaging modalities for the procedure^5^, in rare occasions similar to our case, TTE can be used as a reliable alternative.

When compared with surgical closure, TVSDC indications are evolving constantly; they are the muscular VSD, patients with a high preoperative risk, iatrogenic defects associated with the surgical aortic valve replacement or septal myectomy in HCMP and residual leaks after the surgical VSD closure.^3^ Although, the transcatheter technique for perimembranous VSD closure is not approved by the FDA in the USA, it is widely used in European centers.^5^,^6^ Patient selection for muscular TVSDC is crucial to preventing any predictible complication such as device malopposition, migration or embolization. If the defect proximity to all valves is less than 4 mm, acute endocarditis is detected, and developed Eisenmenger physiology is present, TVSDC is contraindicated.^5^,^7^ In addition, surgical VSD closure should be strongly considered in the patients with VSD concomitant with complex congenital anomalies such as right ventricular outflow obstruction, double outlet right ventricle, severe valve defects etc.^3^,^5^,^7^ Among the most common complications, arrhythmia can be evaluated first. According to Butera G et al.,^8^ complete heart blocks requiring pacemaker were found in 5.7% patients who underwent perimembranous TVSDC. However, Tanidir IC et al.^6^ have reported that 1% patients underwent both perimembraonus and muscular TVSDC. Although the data on this subject are contentious, it can easily be said that fatal conduction disturbances are rare. Hemolysis, device migration or embolization, and pericardial tamponade due to a ruptured, free ventricular wall can be counted as the rare complications.

When using TTE intraoperatively, the most common challenge is obtaining the optimal image of the VSD to maintain and secure the alignment of the occluder device. In our case, we performed the procedure mostly with the A4C view. The advantage of this approach is that the operator can visualize the defect localization and anatomic alignment of the device. However, there are 2 limitations. First, the ultrasound probe can obstruct the fluoroscopic image area, especially in biplane image rendering. To overcome this limitation, the probe should locate more laterally on the chest wall. Another potential solution is obtaining another imaging plane such as the subcostal view to visualize and quantify the defect. Second, suboptimal imaging with the TTE because of technical insufficiencies such as thorax wall malformation, supine position of the patient or an inexperienced sonographer is another major limitation in performing the procedure. Moreover, 3-dimensional imaging of the VSD with a multiplane TTE probe is a relatively new concept; yet an optimal 2-dimensional image should be obtained to render a high-quality 3D image, which is a challenging concept as well.

According to a study which was conducted in infants, TTE-guided VSD closure is feasible with similar success rates compared to TEE-guided procedures.^9^ In another study involving the pediatric and adult age groups, TTE was the imaging choice in 75% of the all TVSDC patients.^6^ However, patients with muscular VSD comprised only 14%, and more importantly, patients older than 16 years constituted only 8% of the cases included in the study.^6^ It is difficult to conclude that TTE is the preferred modality in adult cases with muscular VSD. Therefore, we believe our case is important to prove that the procedure can be performed solely using TTE in adults.

One of our limitations is that although the standard approach for overcoming insufficient image quality with conventional imaging modalities is using ICE, we could not use it for this patient because of the patient’s economic issues.

### Conclusion

Although the majority of VSDs close spontaneously, TVSDC is a reliable option for symptomatic patients with suitable anatomy in adult age. In patients with poor TEE images, TTE can safely and sufficiently guide the percutaneous VSD closure procedure.

## Figures and Tables

**Table 1:** Right Heart Catheterization and Shunt Calculations

O2%	Area	Pressures (mm Hg)			Mix venous O2%:	

		Systolic	Diastolic	Mean		
	SVC				Cardiac output (L/min):	5.87
	IVC				Cardiac index (L/min/m2):	2.81
	RA top	21	18	10	Pulmonary output (L/min):	9.48
71	RA mid				Effective pulmonary output:	6.49
	RA base				Left-to-right shunt (L/min)	3
79	Right ventricle input	42	9		Right to left shunt (L/min)	-
	Right ventricle output				Qp/Qs	1,61
77	Pulmonary artery	42	18	26		
90	Pulmonary artery wedge pressure			13		
92	Aorta	130	80	96		
Transpulmonary gradient (mm Hg):			13		Indexes	
Diastolic pressure gradient (mm Hg)			5		PVR index (Wood Unit dyn.cm.sn^−5^.m^2^)	2.8
PVR (Wood Unit dyn.cm.sn^−5^)			1.4		SVR index (Wood Unit dyn.cm.sn^−5^.m^2^)	29
SVR (Wood Unit dyn.cm.sn^−5^)			14.5		PVRi/SVRi	0.09

SVC: Superior Vena Cava, IVC: Inferior Vena Cava, Qp: Pulmonary output, Qs: Systemic output, PVR: Pulmonary vascular resistance, SVR: Systemic vascular resistance, PVRi: Pulmonary Vascular Resistance Index, SVRi: Systemic Vascular Resistance Index, RA: right artrium.
